# Comparative Pharmacokinetic Assessment of Curcumin in Rats Following Intratracheal Instillation Versus Oral Administration: Concurrent Detection of Curcumin and Its Conjugates in Plasma by LC-MS/MS

**DOI:** 10.3390/pharmaceutics16111459

**Published:** 2024-11-15

**Authors:** Nan Li, Jinle Lou, Lingchao Wang, Wenpeng Zhang, Chunmei Jin, Xiaomei Zhuang

**Affiliations:** 1 Beijing Institute of Pharmacology and Toxicology, Beijing 100850, China; 13345085440@163.com (N.L.); 15665741174@163.com (J.L.); wanglingchao624@163.com (L.W.); wpzhang@bmi.ac.cn (W.Z.); 2 College of Pharmay, Yanbian University, Yanji 133000, China; cmijin@ybu.edu.cn

**Keywords:** LC-MS/MS, curcumin, curcumin–glucuronide conjugate, curcumin–sulfate conjugate, oral, inhalation administration

## Abstract

Objective: To establish and validate an LC-MS/MS method for the simultaneous determination of curcumin (CUR) as well as its glucuronide conjugate (COG) and sulfate conjugate (COS) in rat plasma. The method was employed to evaluate and compare the pharmacokinetic behaviors of curcumin following oral and intratracheal administration in rats. Methods: Rat plasma samples were separated by chromatography on a C18 column after protein precipitation with acetonitrile. Gradient elution with a mobile phase of 0.5 mM ammonium acetate in acetonitrile was utilized. Mass spectrometry detection incorporated an electrospray ionization (ESI) source, multiple reaction monitoring (MRM), and dual-mode (positive and negative) scanning for quantitative analysis. A total of 12 SD rats were randomly divided into two groups and were orally (20 mg/kg) or intratracheally (10 mg/kg) administrated curcumin, respectively. CUR, COG, and COS concentrations in plasma were measured to assess pharmacokinetic disparities. Results: The method demonstrated linearity within the ranges of 2–400 ng/mL for CUR and COS and 5–1000 ng/mL for COG. Intratracheal administration significantly elevated CUR plasma concentrations compared to oral administration. The exposure of COG was higher than COS following oral administration. Conversely, intratracheal administration resulted in markedly higher COS exposure, with no significant difference in COG exposure after dose normalization between oral and inhalation routes. Conclusions: The established LC-MS/MS method provides a reliable tool for the simultaneous measurement of CUR, COG, and COS in rat plasma, facilitating preclinical pharmacokinetic investigations. The study reveals distinct pharmacokinetic profiles for CUR following oral versus intratracheal administration, suggesting that inhalation may offer superior therapeutic efficacy.

## 1. Introduction

Curcumin, a naturally occurring polyphenol derived from the rhizome of turmeric, has attracted considerable interest across various domains such as cosmetics, culinary arts, and pharmaceuticals. Extensive in vivo and in vitro pharmacological studies have demonstrated its diverse therapeutic properties, including its anti-inflammatory effect [1,2,3,4,5,6], as well as its anti-cancer and immunomodulatory activities [7], anti-fibrosis effect [8], antibacterial properties [9], and antioxidant capabilities [10]. Despite these promising pharmacological activities, the development of curcumin as a pharmaceutical agent has been hindered by its unstable physicochemical properties. Its extreme hydrophobicity, sensitivity to light, and metabolic instability pose significant challenges in its formulation and therapeutic application. These factors have limited its translation from a traditional remedy to a mainstream drug. However, ongoing research continues to explore innovative strategies to overcome these obstacles and unlock curcumin’s full potential in modern medicine.

Previous studies have indicated that curcumin exhibits poor permeability (P_app_ = 3.18 ± 1.08 × 10^−6^ cm/s in a Caco-2 cell model) [11]. This characteristic, coupled with a less than favorable pharmacokinetic profile, has significantly limited its oral absorption. Clinical studies have revealed that curcumin was undetectable in the serum of volunteers even after high oral doses of 12 g/day [12]. Similarly, the bioavailability of oral curcumin in rats is less than 1% [13], underscoring the compound’s low systemic availability. The primary reason for the low plasma concentration of curcumin following oral intake is attributed to its extensive in vivo metabolism. Both phase I and phase II metabolizing enzymes are involved in the biotransformation of curcumin. The phase I metabolism, primarily catalyzed by alcohol dehydrogenase present in the liver and intestinal cytoplasm, results in the transformation of metabolites such as 4-hydrocurcumin, 6-hydrocurcumin, and 8-hydrocurcumin. In phase II metabolism, there are two predominant pathways for curcumin metabolism. The first pathway leads to the formation of the glucuronic acid conjugate, known as COG, under the catalytic activity of liver UGT1A1, intestinal UGT1A8, and UGT1A10 [14]. The second pathway involves the transformation to the sulfuric acid conjugate, COS, facilitated by the action of SULT1A1 and SULT1A3 enzymes [14].

In order to enhance the stability and blood concentration of orally administered curcumin, researchers have explored various formulations improvements, such as the development of nanocrystals [15], liposomes, and phospholipid micelles [16]. Despite these efforts, none have achieved the significant expected outcomes. However, by altering the administration route, researchers have discovered that enhanced efficacy could be achieved in animal experiments. Notably, the preparation of curcumin in inhaled formulations has notably improved therapeutic effects in conditions such as lung cancer [17,18,19], pneumonia induced by bleomycin or LPS [20], radiation pneumonitis [21], and even interstitial lung fibrosis. Inhalation administration, being a non-invasive drug delivery method, can directly target therapeutic agents to the respiratory tract and lungs. This approach benefits from the large surface area of the lungs and their abundant blood supply, offering both localized and systemic treatment advantages [22]. However, to date, there have been no reports on the pharmacokinetics of intratracheal administration of curcumin. To fill this research gap, we conducted a comparison of the pharmacokinetic profiles of curcumin in rats following oral and inhalation administration. Utilizing a well-established method, we were able to simultaneously quantify CUR, COS, and COG in rat plasma. This study aims to provide valuable scientific insights for a better understanding and further development of curcumin as a therapeutic agent.

## 2. Materials and Methods

### 2.1. Materials and Reagents

Curcumin (purity > 98%) was purchased from Beijing Inokai Science and Technology Co., Ltd. (Beijing, China). The sulfate conjugate of curcumin (purity > 98%) was purchased from Shanghai Zzbio Co., Ltd. (Shanghai, China). The glucuronide conjugate of curcumin (purity > 98%) was obtained from Shanghai Haoyuan Biomedical Technology Co., Ltd. (Shanghai, China). Curcumin-d6 (purity > 95%), serving as an internal standard of CUR, was acquired from Shanghai Yuanye Biotechnology Co., Ltd. (Shanghai, China). Glipizide (purity > 98%), serving as an internal standard of COG, was purchased from Beijing Bailingwei Technology Co., Ltd. (Beijing, China). Toluenesulfonbutamide (purity > 99%), serving as an internal standard of COS, was purchased from the China Pharmaceutical and Biological Products Inspection Institute (Beijing, China). DMSO (purity > 99.7%) was obtained from Sigma-Aldrich (St. Louis, MO, USA). Acetonitrile (chromatographically pure) was purchased from Thermo Fisher (Waltham, MA, USA). Ammonium acetate (chromatographically pure) was purchased from Sinopharm Group Co., Ltd. (Shanghai, China). Pure water was purchased from Hangzhou Wahaha Group Co., Ltd. (Hangzhou, China). The laryngoscope was purchased from Beijing Huironghe Technology Co., Ltd. (Beijing, China). The rat fixation table was purchased from Shanghai Yuyan Scientific Instrument Co., Ltd. (Shanghai, China).

### 2.2. Experimental Animals

Male Sprague-Dawley rats at a weight of 180~220 g were purchased from the Beijing Sprague-Dawley Laboratory Animal Technology Co. (Beijing, China). These animals were housed under appropriate environmental conditions (temperature: 22 ± 2 °C, relative humidity: 45–60%) for an acclimatization period exceeding 3 days prior to the commencement of experiments, with ad libitum access to food and water. All procedures involving animals were reviewed and approved by the Institutional Animal Care and Use Committee (IACUC) of the Beijing Institute of Pharmacology and Toxicology, with the approval number IACUC-DWZX-2023-P716.

### 2.3. Chromatographic Analysis Methods

A CERIL-column C18 (2.1 mm × 50 mm, 2 μm) was utilized. The mobile phase was a mixture of 0.5 mM ammonium acetate and acetonitrile. The flow rate was set at 0.4 mL per minute. The gradient elution program was as follows: (0–0.3 min, 20% B; 0.3–2.5 min, increasing to 95% B; 2.5–3 min, maintained at 95% B; 3–4.01 min, returned to 20% B). The column temperature was maintained at 40 °C, and the sample injection volume was 7 μL.

### 2.4. Mass Spectrometry Analysis Conditions

LC-MS/MS 8050 (Shimadzu Company, Kyoto, Japan), equipped with a selective electrospray ion (ESI) source and operating in multi-reaction monitoring (MRM) mode, was employed to quantify CUR, COS, and COG by integrating the scanning of negative and positive ion modes. The mass spectrum detection parameters for CUR, COS, and COG and their respective internal standards CUR-D6, toluenbutamide (TB), and glipizide (GLI) are shown in Table 1. The secondary mass spectra of CUR, COS, and COG and CUR-D6, TB, and GLI are depicted in Supplementary Figure S1.

### 2.5. Preparation of Stock and Working Solution

Each reference standard was accurately weighed to prepare the stock solutions in DMSO at a concentration of 10 mg/mL for CUR, COG, and COS. A cocktail of standard working solutions was obtained by sequential dilution with ACN. The plasma concentrations for CUR and COS were established at 2, 5, 10, 20, 50, 100, 200, and 400 ng/mL, while COG concentrations were set at 5, 12.5, 25, 50, 125, 250, 500, and 1000 ng/mL. A mixed internal standard (IS) solution containing CUR-D6 at 25 ng/mL, TB at 10 ng/mL, and GLI at 10 ng/mL was also prepared using the aforementioned method.

Double blank plasma samples (DB) and plasma samples containing internal standard only (CB) were prepared. Quality control (QC) sample working solutions were formulated, with low, medium, and high concentrations for CUR and COS in rat plasma set at 6, 60, and 320 ng/mL, respectively. Correspondingly, the concentrations for COG in rat plasma were 15, 150, and 800 ng/mL.

### 2.6. Sample Processing

Rat plasma samples were processed using the acetonitrile protein precipitation method. A 20 μL aliquot of rat plasma sample was pipped and added with 20 μL of acetonitrile and 200 μL of acetonitrile containing the mixed internal standard. The mixture was then vortexed for 1 min to ensure thorough mixing. Following this, the sample was centrifuged at 14,000× *g* for 10 min to separate the protein precipitate from the supernatant. The supernatant, which contains the analytes of interest, was carefully transferred to an injection vial for subsequent LC-MS/MS analysis.

### 2.7. LC-MS/MS Method Verification

#### 2.7.1. Specificity and Selectivity

In order to ascertain the specificity and selectivity of the method, a comparative analysis of chromatograms for each analyte was conducted using pooled blank plasma from six rats, spiked blank plasma samples, and the actual plasma samples post-administration.

#### 2.7.2. Matrix Effect and Extraction Recovery

In a separate test for matrix effects, rat plasma was substituted with pure water. The peak area of CUR, COS, and COG was measured, and the peak area ratio of these compounds in the plasma sample to that in the pure water sample was determined after sample preparation. This step assists in evaluating potential ion suppression or enhancement effects that could occur during the analysis. Subsequently, another 20 μL of the mixed plasma was treated with 20 μL of the QC working solution, encompassing low (LQC), medium (MQC), and high (HQC) concentrations, both before and after protein precipitation. The peak areas of CUR, COS, and COG were detected at these stages, and the extraction recovery was calculated based on the ratio of the peak areas before and after the protein precipitation step. This calculation is crucial for understanding the efficiency of the sample preparation process and the potential for matrix interferences in the analytical method.

#### 2.7.3. Linearity Range and Lower Limit of Quantification

The linearity of the method was assessed by analyzing a series of samples with known concentrations. A weighting factor of 1/X^2^ was applied to account for potential curvature in the calibration plot. The least squares regression fitting was performed using the series of concentrations of the compounds to generate a calibration curve. The concentration of the analyte in the quality control (QC) or test samples was determined from the regression parameters derived from the calibration curve. The precision and accuracy of the lower limit of quantification (LLOQ) samples, which should exhibit a signal-to-noise ratio greater than or equal to 10, were evaluated. The relative error (RE) should not exceed 20% for LLOQ samples, indicating that the method is reliable and reproducible at the lowest concentration level of the calibration range. This ensures that the method is suitable for detecting and quantifying the analytes of interest in the biological samples with confidence.

#### 2.7.4. Precision and Accuracy

CUR and COS were prepared in rat plasma to make LLOQ, LQC, MQC, and HQC (2, 6, 60, and 320 ng/mL) samples (*n* = 6), and COG was prepared in rat plasma to make LLOQ, LQC, MQC, and HQC (5, 15, 150, and 800 ng/mL) samples (*n* = 6). Three analyses were performed in three independent analytical batches (*n* = 18), accompanied by the fresh preparation of a calibration curve to assess intra-batch and inter-batch precision (RSD) and accuracy (RE). The validation criteria were RSD ≤ 20% and RE within ± 20% for LLOQ and RSD ≤ 15% and RE within ± 15% for the remaining QC samples.

#### 2.7.5. Stability

The stability of rat plasma samples spiked at LQC, MQC, and HQC concentration (*n* = 6) was investigated under conditions that simulated various storage scenarios. These included storage at room temperature for 4 h, at 4 °C for 8 h, at −40 °C for 7 days, and the experience three freeze–thaw cycles between −40 °C and room temperature.

#### 2.7.6. Dilution Linearity

A rat plasma sample with a high concentration of CUR at 2000 ng/mL was prepared using blank rat plasma (*n* = 5). To assess the dilution linearity, these samples were diluted with a 9-fold volume of blank rat plasma to achieve a final concentration of 200 ng/mL before processing.

### 2.8. Pharmacokinetic Study

Curcumin was formulated into a self-emulsifying preparation at a concentration of 200 mg/mL using a self-emulsifying preparation composed of 15% isopropyl cinnamate, 35% polyoxyethylene 35 castor oil, 35% polyethylene glycol glyceryl caprylate, and 15% diethylene glycol monoethyl ether. This preparation was then diluted with saline to obtain the desired concentrations for animal experiments.

**Figure 1:**
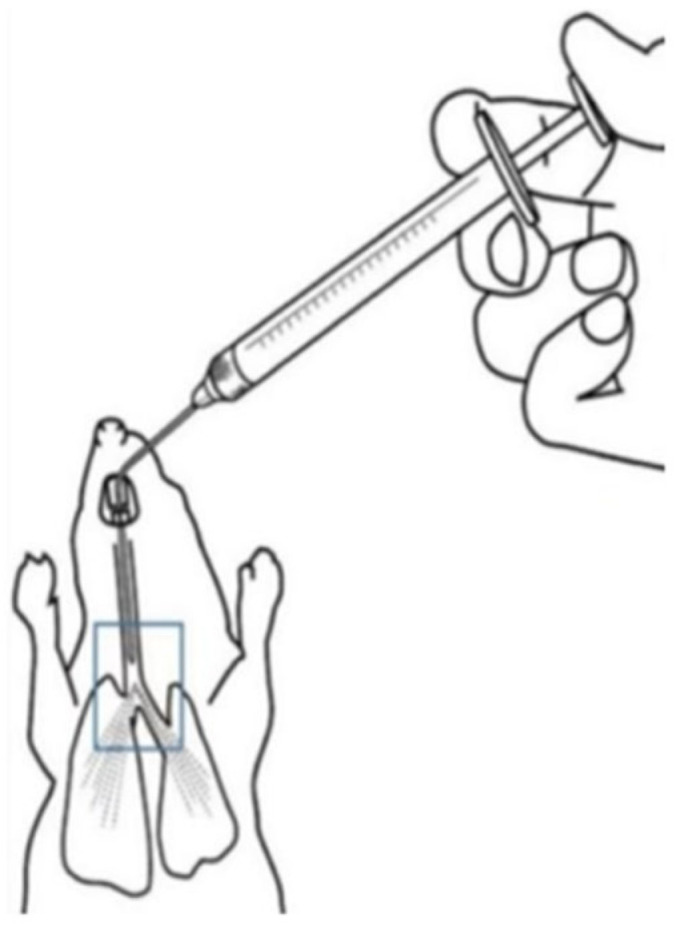
Schematic diagram of intratracheal instillation.

Twelve male SD rats were randomly divided into two groups with six rats each, one for oral administration and the other for inhalation administration. The oral administration group received a single intragastric dose of 20 mg/kg curcumin. Blood samples of approximately 200 μL were collected via intravenous routes at pre-determined time points: before administration and at 5, 15, 30, and 45 min, and 1, 2, 4, 6, and 8 h post-administration. The blood samples were immediately placed in heparinized EP tubes and kept on ice. For the inhalation group, the rats were administered 10 mg/kg curcumin via tracheal inhalation. The specific method of intratracheal administration is depicted in Figure 1, and details of the administration process are provided in the Supplementary Materials (Supplementary Figure S2). Blood samples of about 200 μL were collected at time points before administration and at 2, 5, 15, and 30 min, and 1, 2, 4, 6, 8, 12, and 24 h post-administration, also placed in heparinized EP tubes and kept on ice. The whole blood samples were centrifuged at 4 °C at 5000× *g* for 10 min within 30 min of collection. The plasma was then separated, aliquoted, and stored at −40 °C until analysis.

### 2.9. Data Analysis and Processing

The major pharmacokinetic parameters including elimination half-life (t_1/2_), time to reach peak concentration (T_max_), peak concentration (C_max_), area under the curve (AUC), and mean residence time (MRT) were calculated using Phoenix WinNonlin software (Version 8.2, Certara, Radnor, PA, USA). A non-compartmental model was employed for these calculations, which is suitable for analyzing drug concentration data over time without assuming a specific compartmental structure. Graph Prism 8 was utilized to create graphs and plots that illustrate the pharmacokinetic behavior of the compounds of interest. Statistical analysis was performed using IBM SPSS Statistics 26 to conduct a *t*-test on the dose-normalized AUC_(0−t)_ values from the two different administration groups. This analysis aimed to determine the differences in the in vivo exposure of CUR, COS, and COG in rats following oral and inhalation administration. The results were considered statistically significant at a *p*-value less than 0.05, indicating a difference between the groups. A *p*-value less than 0.01 was considered to represent a highly significant statistical difference, suggesting a more pronounced distinction in the pharmacokinetic profiles of the treatments compared.

## 3. Results

### 3.1. Method Development

To achieve the most sensitive mass spectrometry parameters for the simultaneous detection of curcumin and its conjugates, we compared signal intensities of equivalent concentrations of CUR and conjugates in both positive and negative ion modes. The finding indicated that higher intensities for CUR and COS were obtained in negative ion mode, whereas COG showed superior intensity in positive ion mode. Consequently, the method was established to scan both positive and negative ion modes simultaneously. After optimizing with various compounds as internal standards, CUR-D6 was selected for CUR, tetrahydrobiopterin (TB) for COS, and glibenclamide (GLI) for COG, with monitored transitions set at 367.15→134.1 for CUR, 447.00→134.1 for COS, 545.15→369.15 for COG, and 373.15→369.15 for CUR-D6. To refine the mobile phase gradient, methanol and acetonitrile were compared as organic solvents, with the acetonitrile/water system demonstrating greater sensitivity and sharper peaks. The addition of 0.5 mM ammonium acetate to the aqueous phase was found to enhance the compound ionization and further improve the peak shapes. Following a comparison of several C18 chromatographic columns, the CERIL-column C18 (2.1 mm × 50 mm, 2 μm) was chosen for its optimal peak shape and separation.

### 3.2. Plasma Sample Process Method

For plasma sample processing, we sought a straightforward and efficient method, initially attempting protein precipitation using organic solvents. A comparison between methanol and acetonitrile as precipitants showed that acetonitrile yielded superior precipitation efficiency, cleaner sample processing, and enhanced sensitivity. Utilizing an 11-fold volume of acetonitrile for protein removal in rat plasma samples ensured minimal matrix effects while maintaining sensitivity.

### 3.3. Method Verification

#### 3.3.1. Selectivity

**Figure 2:**
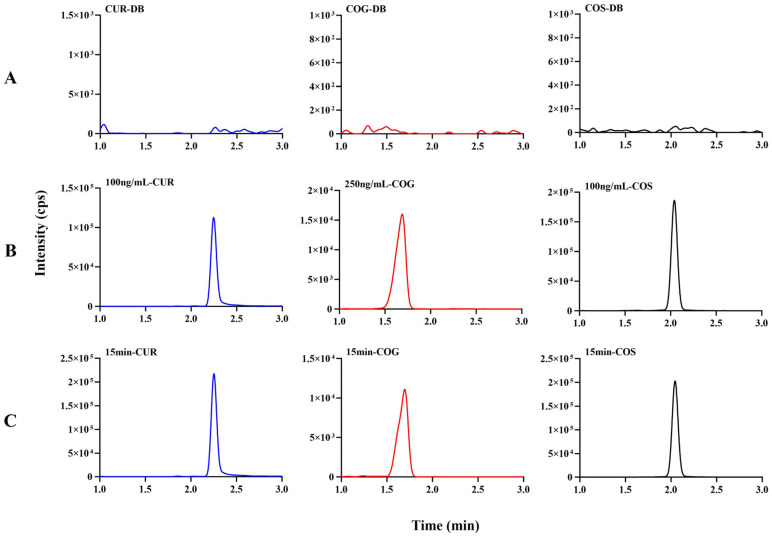
Representative chromatograms of CUR, COS, and COG in rat plasma samples. (**A**) Rat blank plasma. (**B**) Rat plasma spiked with 100 ng/mL CUR, 100 ng/mL COS, and 250 ng/mL COG. (**C**) Actual sample taken from rat after CUR (10 mg/kg) was administered by inhalation at 15 min.

As depicted in Figure 2, under the assay conditions, all analytes displayed well-shaped chromatographic peaks with complete baseline separation. The chromatographic retention times of COG, COS, and CUR were 1.6 min, 1.7 min, and 2.2 min, respectively. No rat plasma endogenous substance interfered with the detection of each compound of interest at the respective retention times.

#### 3.3.2. Matrix Effect and Extraction Recovery

The method recoveries of CUR, COS, and COG in rat plasma exceeded 95.3%. A slight matrix effect was observed for COG in rat plasma, ranging from 82.2 to 85.8%. The variability was within 2.8–5.2%, which did not impact the quantitative accuracy of the samples. CUR and COS showed no obvious matrix effects. Detailed data are shown in Table 2.

#### 3.3.3. Linear Range and Lower Limit of Quantification

Plotting the concentrations of CUR, COS, and COG X (ng/mL) against the peak ratio to their respective internal standards, regression analysis was carried out using a weighted (1/X^2^) least squares method. This yielded the following regression equations for plasma: for CUR, Y = 0.00250120X − 0.000259836 (2–400 ng/mL); for COS, Y = 0.0133826X − 0.003889490 (2–400 ng/mL); and for COG, Y = 0.00143620X − 0.00301771 (5–1000 ng/mL). All the compounds demonstrated a linear correlation exceeding 0.99, indicating a robust linear relationship between concentration and response.

#### 3.3.4. Precision and Accuracy

As shown in Supplementary Table S1, the inter-batch and intra-batch precision of all the QC samples in CUR, COS, and COG rat plasma were all within 11.2%, and the accuracy was within ±17.4%, which displayed that the methodology met the requirements.

#### 3.3.5. Stability

The accuracies and precisions of CUR, COS, and COG in spiked rat plasma QC samples, after being stored at room temperature for 4 h, at 4 °C for 8 h, at −40 °C for 7 days, and subjected to three freeze–thaw cycles between −40 °C and room temperature, were all within ±14% and 7.3% of each target compound, respectively. It showed that the rat plasma samples containing CUR, COS and COG were maintained as stable under the aforementioned storage conditions. Detailed data are shown in Supplementary Table S2.

#### 3.3.6. Dilution Linearity

The accuracy of plasma samples containing a high concentration of CUR remained within ±3% after being diluted 10-fold to 200 ng/mL by blank rat plasma. This demonstrates the reliability of the dilution process for samples with concentrations exceeding the assay’s calibration range.

### 3.4. Pharmacokinetic Study

**Figure 3:**
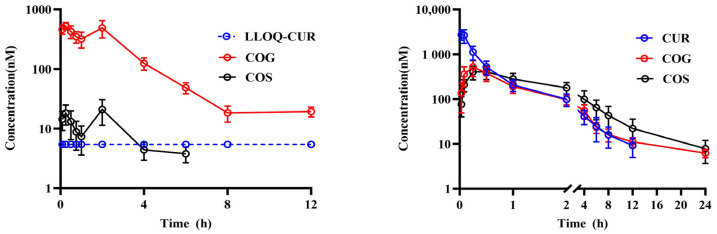
Average concentration–time curves of plasma curcumin and its conjugates after oral administration of 20 mg/kg (**left**) and inhalation of 10 mg/kg curcumin (**right**) in rats (*n* = 6) (blue dotted line is the lower limit of quantification of CUR).

The concentration–time curves and pharmacokinetic parameters of CUR, COG, and COS after oral and inhalation of CUR in rats are depicted in Figure 3 and Table 3. The findings indicate that after oral dosing of 20 mg/kg curcumin, the plasma levels of the parent compound CUR were below the lower limit of quantification (2 ng/mL = 5.434 nM). In contrast, the metabolites COG and COS were detectable, with COG exhibiting an exposure 30–40 times greater than COS. Upon inhalation of 10 mg/kg curcumin, the drug directly targeted the lungs and was rapidly absorbed into the systemic circulation, resulting in plasma concentrations of CUR significantly higher than those achieved via oral administration. Notably, COS concentrations were substantially higher in the inhalation group compared to the oral group, while COG concentrations were comparable when normalized for dose (inhalation administration/0.5).

**Figure 4:**
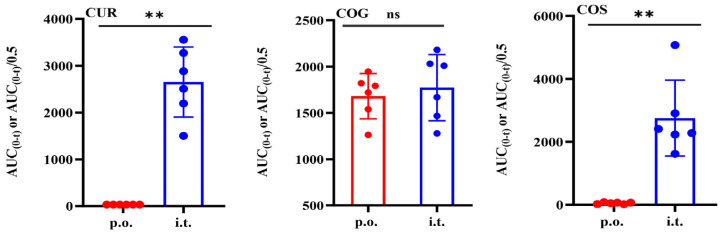
Comparison of dose-normalized curcumin and its conjugates in plasma after oral (20 mg/kg) and intratracheal administration (10 mg/kg) of curcumin in rats (inhalation/0.5) (*n* = 6). **: *p* < 0.01 statistically significant difference compared to oral administration and ns: not statistically significant.

To accurately assess the impact of the two administration routes on curcumin’s pharmacokinetics, the areas under the curve (AUC) were compared after dose normalization (inhalation dose/0.5). As depicted in Figure 4, inhalation led to a marked increase in CUR exposure from below the detection limit to an AUC_(0–t)_/0.5 of 2652 ± 748.8 h·nM. The exposure of COG, with an AUC_(0–t)_/0.5 of 1772 ± 356.6 h·nM, was not significantly different from oral administration (AUC_(0–t)_ of 1680 ± 244.1 h·nM). However, the exposure of COS, with an AUC_(0–t)_/0.5 of 2754 ± 1208 h·nM, was nearly 50-fold higher than that of oral administration (AUC_(0–t)_ of 49.78 ± 27.14 h·nM). This disparity may be attributed to the higher activity of sulfotransferase (SULT) enzymes in the lungs compared to the intestine and liver.

## 4. Discussion

Researchers have found that intratracheal administration can alter the course of the drug in the body, and direct administration of curcumin through the respiratory tract may overcome the first-pass effect of curcumin, where the effects of intestinal absorption, intestinal metabolism, and liver metabolism are avoided, potentially enhancing its efficacy, especially for the treatment of pulmonary diseases [22]. This paper presents, for the first time, a plasma pharmacokinetic study in rats following curcumin inhalation via the respiratory tract compared to oral administration.

A robust quantitative assay method for the determination of target compounds in plasma samples is essential for pharmacokinetic studies. In order to thoroughly characterize the pharmacokinetic behavior of curcumin, a method for the simultaneous detection of curcumin and its conjugated metabolites COG and COS in rat plasma was established. Although numerous methods for quantifying curcumin in biological matrices have been reported, the complexity of curcumin biotransformation has made LC-MS/MS the predominant approach in recent years for supporting pharmacokinetic studies of curcumin. According to a review of LC-MS/MS methods for curcumin and its metabolites by Kotha and Luthria et al. [23], most assays for curcumin conjugates rely on β-glucuronidase hydrolysis, which breaks down the conjugates to the parent compound for indirect assay. This method is cumbersome, and the hydrolysis conversion rate cannot be guaranteed to reach 100%. In addition, most β-glucuronidases lack specificity and cannot differentiate between sulfate and glucuronide conjugates.

In this study, a method for simultaneous scanning of positive and negative ions for the detection of curcumin, glucuronide conjugate, and sulfate conjugate was established, with the analysis time of each sample only being 4 min to achieve the complete baseline separation of the peaks, enabling high-throughput detection. Meanwhile, due to the complex composition of the biological samples, it is crucial to select a biological sample pre-treatment method that effectively removes proteins and impurities. After repeated optimization, it was finally determined that rat plasma samples being treated with 11-fold volume acetonitrile precipitation for protein precipitation is a more time- and cost-efficient method compared to most liquid–liquid extraction and solid-phase extraction methods reported in the literature [24,25]. This approach has been validated to essentially eliminate the matrix effect of samples and is capable of detecting plasma samples after curcumin administration. However, the limitation of this method is that only an LC-MS/MS method for curcumin and its conjugates was established in this study to investigate the pharmacokinetic characteristics of curcumin and its conjugates in rat plasma after the oral and inhalation administration of curcumin. The pharmacokinetic characteristics of its reduction metabolites in rats were not considered.

After oral administration of 20 mg/kg curcumin to rats, in agreement with previous study reports [14], the plasma curcumin concentration was extremely low (below the detection limit 2 ng/mL). The primary metabolites present were COG and COS, with the content of COG being significantly higher than that of COS. A characteristic double-peak phenomenon was observed in the blood concentration–time profiles for both COG and COS. After oral administration, curcumin undergoes metabolism to form COG and COS, which have enhanced water solubility. These metabolites are then excreted into the intestines through bile, where they are hydrolyzed by intestinal flora and enzymes to release the parent compound. Subsequently, the released curcumin is absorbed by the intestinal tract, transported to the liver, and further metabolized back into COG and COS.

Given that the animals were incapable of actively inhaling the drug, intratracheal administration was employed to mimic the process of inhalation administration in this study. Due to the limitations in curcumin’s solubility and the volume of the intratracheal administration, the dosage of intratracheal instillation curcumin for rats was set as 10 mg/kg. At this half oral dosage, the plasma concentrations of CUR, COG, and COS were measurable after the rats’ intratracheal administration of curcumin. The rate at which CUR was absorbed into the bloodstream through the lungs was very rapid. Conversely, the absorption rates of COG and COS into the bloodstream were slower than those following oral administration but faster than the rates observed with oral intake. Secondly, within 0.5 h after intratracheal administration, CUR reached the highest blood concentration, with COG and COS concentrations being lower than that of CUR. After 0.5 h, the elimination rate of COS notably decreased, resulting in comparable overall exposure for CUR and COS, while the exposure for COG remained low.

This study demonstrates for the first time that the metabolic pathway of curcumin through the lungs is significantly different from that of the intestine, suggesting that intratracheal administration could be a highly effective method of delivery. The exposure characteristics of the parent drug and its metabolites at pulmonary sites and systemic tissues following curcumin intratracheal instillation warrant further investigation.

## 5. Conclusions

In summary, a highly sensitive LC-MS/MS method was developed and validated for quantitative determination of CUR, COG, and COS concentrations in rat plasma, aligning with the ICH-M10 guidelines (European Medicines Agency, EMA). Pharmacokinetic studies in rats following oral and intratracheal administration of curcumin using this method revealed significant differences in the exposure characteristics of these compounds. Notably, following intratracheal instillation, the concentration of CUR was considerable higher than that after oral administration. When exposures of COG were compared between oral and intratracheal administration, they were found to be similar after adjusting for the dose. However, the plasma concentrations of COS were observed to be higher when administered via intratracheal instillation compared to oral administration. The findings suggest that administering curcumin via intratracheal instillation may represent an important future research direction, potentially increasing the bioavailability of curcumin and enhancing its therapeutic efficacy.

## Figures and Tables

**Table 1:** Mass spectrometry detection conditions for CUR, COS, and COG and internal standard CUR-D6, TB, and GLI.

Compound	*m*/*z*		CE (V)	Q1 (V)	Q3 (V)
	Precursor Ion	Product Ion			
CUR	367.15	134.10	−32	−20	−28
COS	447.00	134.10	−42	−24	−14
COG	545.15	369.15	16	20	17
CUR-D6	373.15	134.10	−32	−20	−14
TB	269.40	169.90	−17	−20	−17
GLI	446.30	321.15	15	10	15

**Table 2:** Recoveries and matrix effects of CUR, COS, and COG in rat plasma (mean ± SD, *n* = 6).

Compound	Concentration (ng/mL)	Recovery (%)	Matrix Effect (%)	Matrix Effect RSD (%)
CUR	6	98.7 ± 8.0	109.3 ± 3.6	3.3
	60	100.5 ± 7.0	/	/
	320	95.3 ± 5.7	111.0 ± 3.1	2.8
COS	6	100.7 ± 9.3	107.7 ± 5.1	4.8
	60	104.4 ± 7.5	/	/
	320	97.1 ± 6.3	110.2 ± 3.4	3.0
COG	6	146.8 ± 14.1	85.8 ± 4.4	5.2
	60	123.4 ± 10.9	/	/
	320	111.9 ± 5.6	82.2 ± 2.3	2.8

**Table 3:** Main pharmacokinetic parameters of curcumin and its conjugates after oral and intratracheal administration of curcumin in rats (mean ± SD, *n* = 6).

Parameters	Unit	p.o. (54.34 μmol/kg) (20 mg/kg)			i.t. (27.17 μmol/kg) (10 mg/kg)		
		CUR	COG	COS	CUR	COG	COS
t_1/2_	h	/	1.74 ± 0.57	1.11 ± 0.87	3.73 ± 1.03	3.10 ± 0.95 *	5.02 ± 1.27 **
T_max_	h	/	0.54 ± 0.71	1.13 ± 0.96	0.05 ± 0.03	0.25 ± 0.00	0.38 ± 0.14
C_max_	nM	/	829.5 ± 158.4	23.44 ± 9.40	2878 ± 741.5	520.9 ± 203.4	415.8 ± 137.6
AUC_(0–t)_	h·nM	<32.58	1680 ± 244.1	49.78 ± 27.14	1326 ± 374.4	886.3 ± 178.3	1377 ± 604.3
AUC_(0–∞)_	h·nM	/	1730 ± 256.5	57.70 ± 27.78	1376 ± 370.4	918.9 ± 167.8	1444 ± 624.9
MRT_(0–t)_	h	/	2.54 ± 0.29	2.37 ± 0.98	2.01 ± 0.78	4.33 ± 1.69 *	5.14 ± 1.03 **
AUC_(0–t)_/0.5	h·nM	/	/	/	2652 ± 748.8	1772 ± 356.6	2754 ± 1208

Notes: p.o.: oral administration; i.t.: tracheal inhalation administration; Exposure (AUC) for oral prototype was calculated using the lower limit of quantification over 6 h; AUC_(0–-t)_/0.5 was used for intratracheal administration only; * *p* < 0.05 statistically different and ** *p* < 0.01 statistically significant difference compared to oral administration.

## Data Availability

Data will be made available on request.

## Supplementary Materials

The following supporting information can be downloaded at: https://www.mdpi.com/article/10.3390/pharmaceutics16111459/s1, Figure S1: Secondary mass spectra of CUR, COS, COG and their internal standard CUR-D6, TB and GLI; Table S1: Precision and accuracy of the LC-MS/MS method for quantification of CUR, COS, and COG in rat plasma (Mean ± SD, *n* = 6); Table S2: Stability of CUR, COS, COG in rat plasma (Mean ± SD, *n* = 3); Figure S2: Schematic diagram of intratracheal instillation.
