# Supplementary material for: Comparative Pharmacokinetic Assessment of Curcumin in Rats Following Intratracheal Instillation Versus Oral Administration: Concurrent Detection of Curcumin and Its Conjugates in Plasma by LC-MS/MS
Source: Pharmaceutics. 2024 Nov 15;16(11):1459. doi: 10.3390/pharmaceutics16111459 (PMC11597260; doi:10.3390/pharmaceutics16111459)
Supplement: Supplementary file 1 [file pharmaceutics-16-01459-s001.zip › pharmaceutics-3277625-supplementary.pdf]

Supplementary Materials

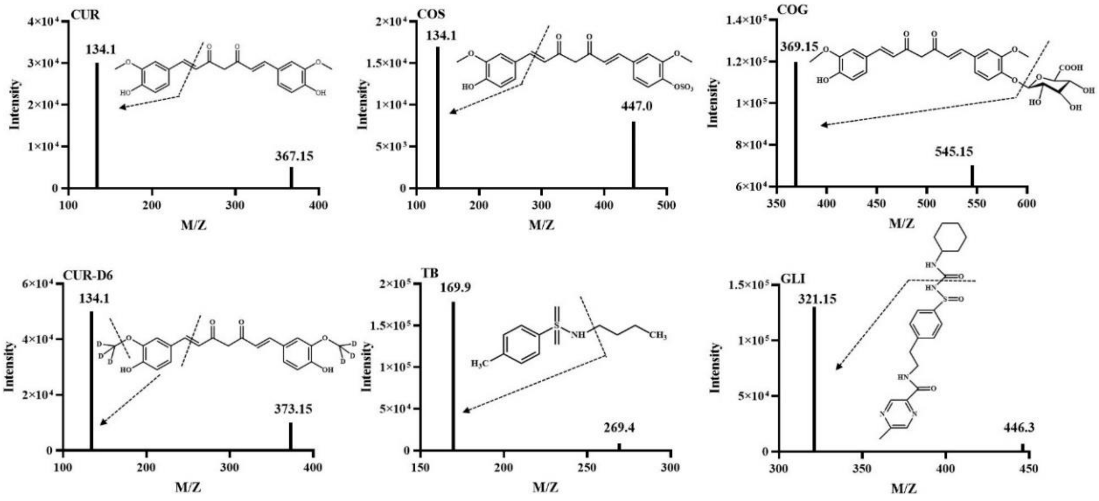

Supplementary Figure S1. Secondary mass spectra of CUR, COS, COG and their internal standard CUR-D6, TB and GLI.

Supplementary Table S1. Precision and accuracy of the LC-MS/MS method for quantification of CUR, COS, and COG in rat plasma (Mean ± SD, *n* = 6).

Compound	Concentration (ng/mL)	Intra-day (n=6)						Inter-day (n=18)	
		RE (%)			RSD (%)			RE (%)	RSD (%)
CUR	2	91.36±5.83	103.13±7.64	109.85±6.43	6.3	6.7	5.9	101.35±10.18	6.5
	6	99.70±2.08	98.97±3.41	99.83±3.51	2.1	3.2	3.5	99.53±2.88	2.8
	60	98.97±3.41	98.64±2.04	97.32±2.41	3.0	2.1	2.5	100.23±4.07	2.6
	320	99.83±3.51	98.42±1.00	96.53±0.95	1.9	1.0	1.0	99.92±3.84	1.8
COS	2	88.07±5.27	102.90±5.63	112.28±4.05	6.0	5.5	3.6	100.76±11.55	11.2
	6	90.79±4.24	95.88±1.32	100.27±3.84	4.5	1.4	3.8	95.63±5.26	5.3
	60	89.25±3.10	94.71±1.13	96.63±1.66	3.5	1.2	1.7	93.45±3.89	4.1
	320	88.03±1.90	93.97±1.58	96.53±1.69	2.2	1.7	1.7	92.56±3.94	4.3
COG	2	113.06±5.62	107.78±9.65	117.31±3.55	5.0	9.0	3.0	112.72±7.51	6.5
	6	95.30±9.19	89.78±5.05	95.12±7.83	9.6	5.6	8.2	93.40±7.57	2.8
	60	93.96±3.71	94.82±5.35	99.52±1.65	4.0	5.6	1.7	96.10±4.43	2.6
	320	97.97±7.35	100.04±5.79	102.77±8.48	7.5	5.8	8.3	100.26±7.14	1.8

Supplementary Table S2. Stability of CUR, COS, COG in rat plasma (Mean ± SD, *n* = 3).

Compound	Concentration (ng/mL)	Room temperature for 4 h		4 ℃ for 8 h		Freeze-thaw three cycles		40 ℃ for 7 d	
		Accuracy (%)	RSD (%)	Accuracy (%)	RSD (%)	Accuracy (%)	RSD (%)	Accuracy (%)	RSD (%)
CUR	6	92.53±2.91	3.2	96.00±2.57	2.7	102.33±7.46	7.3	105.13±4.11	3.9
	60	98.57±2.22	2.2	97.73±0.83	0.9	95.20±2.19	2.3	97.40±0.36	0.4
	320	103.93±1.07	1.0	102.67±0.06	0.1	90.10±4.20	4.6	93.87±3.21	3.4
COS	6	99.67±4.83	4.9	100.63±2.40	2.4	93.73±4.97	5.3	90.73±1.36	1.5
	60	96.63±1.39	1.4	93.27±2.31	2.4	93.23±0.96	1.1	94.53±0.40	0.5
	320	100.77±0.65	0.6	99.23±1.50	1.6	93.80±0.46	0.5	92.43±5.58	6.0
COG	6	90.20±4.16	4.6	88.10±4.59	5.2	89.23±3.79	4.3	104.10±7.03	6.7
	60	95.53±2.01	2.1	90.43±2.97	3.3	87.93±2.81	3.2	87.13±2.10	2.4
	320	95.73±2.23	2.4	93.60±2.52	2.7	87.87±2.32	2.6	88.93±0.99	1.1

Below is the description of the delivery device and the detailed procedure for intratracheal drug delivery.

1. The drug delivery device consists of three main components: a rat fixation table, an animal laryngoscope, and a tracheal drug delivery syringe.

2. The rat fixation table is utilized for securing rats post-anesthesia to ensure that the animal's pharynx is at an appropriate angle for laryngoscopic manipulation. Due to the deep and narrow pharynx of rats and their complex physiological structure, the pharynx cannot be directly visualized with the naked eye. Therefore, it is essential to use the laryngoscope to accurately identify the pharyngeal position. The light-emitting probe on the laryngoscope aids in clearly displaying the pharyngeal tissues, which facilitates the precise intratracheal administration of medication.

3. As depicted in the figure, the anesthetized rats were initially positioned on the rat fixation table. With the assistance of the laryngoscope, the trachea of the rats was exposed, and the tracheal drug delivery needle was carefully inserted into the trachea. Subsequently, the drug was slowly injected into the trachea over a period of 3 seconds.

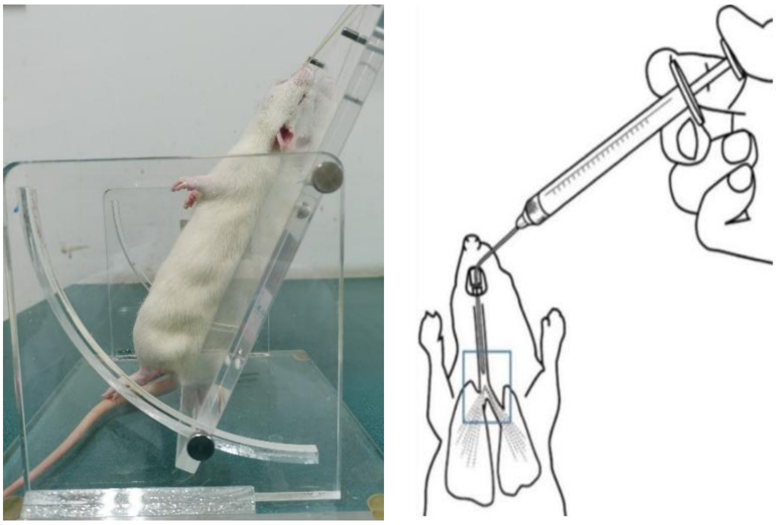

Supplementary Figure S2. Schematic diagram of intratracheal instillation
